# Targeting PDK1: A novel approach to combat hypoxia‐induced epithelial‐mesenchymal transition in chronic rhinosinusitis with nasal polyps

**DOI:** 10.1002/clt2.70048

**Published:** 2025-04-02

**Authors:** Sicen Pan, Mengyan Zhuang, Xiangdong Wang, Qinqin Zhang, Ting He, Ying Li, Jian Jiao, Luo Zhang

**Affiliations:** ^1^ Department of Otolaryngology Head and Neck Surgery Beijing TongRen Hospital Capital Medical University Beijing China; ^2^ Beijing Laboratory of Allergic Diseases Beijing Municipal Education Commission Beijing Key Laboratory of Nasal Diseases Key Laboratory of Otolaryngology Head and Neck Surgery Beijing Institute of Otolaryngology Ministry of Education Capital Medical University Beijing China; ^3^ Department of Allergy Beijing TongRen Hospital Capital Medical University Beijing China

**Keywords:** chronic rhinosinusitis with nasal polyps, epithelial‐mesenchymal transition, hypoxia, lactate, pyruvate dehydrogenase kinase 1

## Abstract

**Background:**

Hypoxia is a prevalent pathological process in chronic rhinosinusitis with nasal polyps (CRSwNP), leading to a cascade of pathological events, including epithelial‐mesenchymal transition (EMT). However, the mechanisms underlying hypoxia‐induced EMT remain unclear. This study aims to elucidate the mechanisms driving EMT under hypoxic conditions in CRSwNP.

**Methods:**

Transcriptome and proteome analyses of hypoxia‐treated human nasal epithelial cells (HNECs) were performed to identify key molecules and pathways. The expression of hypoxia‐inducible factor‐1α (HIF‐1α), pyruvate dehydrogenase kinase (PDK1), lactate dehydrogenase A (LDHA), and EMT markers was assessed in nasal tissues from CRSwNP patients. In vitro, cultured HNECs were exposed to hypoxia and lactate, or overexpressed PDK1, to evaluate changes in EMT markers.

**Results:**

Hypoxia activated the glycolysis‐related pathway in HNECs, with PDK1 and LDHA identified as significantly upregulated glycolysis‐related enzymes. The expression of PDK1 and LDHA was closely correlated with HIF‐1α and EMT markers in nasal tissues. Hypoxia induced an increase in PDK1 and LDHA expression, lactate production, and EMT occurrence in HNECs. PDK1 overexpression or lactate stimulation also triggered EMT, while PDK1 inhibition attenuated hypoxia‐induced EMT in HNECs.

**Conclusions:**

This study is the first to reveal that hypoxia‐induced activation of PDK1 plays a critical role in regulating EMT by promoting lactate production, thereby providing a potential therapeutic target for CRSwNP.

## 1 INTRODUCTION

Chronic rhinosinusitis (CRS) is a persistent inflammation of the nasal cavity and sinus mucosa, characterized by clinical manifestations such as nasal congestion, runny nose, facial pain, and anosmia lasting more than 12 weeks.^1^ CRS can be classified based on the presence or absence of nasal polyps into CRS without nasal polyps (CRSsNP) and CRS with nasal polyps (CRSwNP).^2^ Additionally, CRSwNP can be further divided into eosinophilic CRSwNP (ECRSwNP) and non‐eosinophilic CRSwNP (NECRSwNP) according to the degree of eosinophil infiltration. Nasal polyps are inflammatory lesions that arise from the mucous membranes of the nasal cavity or paranasal sinuses. The mechanisms underlying their formation are complex and involve factors such as superantigen effects, microbial imbalances, biofilm formation, and defects in immune barriers.^3^ Epithelial‐mesenchymal transition (EMT) is a significant pathological feature of CRSwNP, playing a crucial role in the complex remodeling of nasal mucosal tissue and the maintenance of the inflammatory environment in the nasal cavity.^4^ However, the mechanisms by which EMT occurs in CRSwNP remain largely unclear.

Hypoxia is a prevalent pathological process in CRSwNP. This condition is often accompanied by nasal anatomical abnormalities and the presence of nasal polyps, which can lead to nasal sinus stenosis, further impairing air circulation. Coupled with inflammation, infection, and other contributing factors, the nasal tissues and cells of CRSwNP patients experience varying degrees of hypoxia. Decreased oxygen levels upregulate the expression of the transcription factor hypoxia‐inducible factor‐1α (HIF‐1α), which is highly expressed in the nasal mucosa of CRSwNP patients.^5^ The hypoxia and HIF‐1α pathway has been identified as one of the key signaling pathways regulating the EMT process in CRSwNP^6^; however, the precise mechanism by which hypoxia/HIF‐1α regulates EMT in CRSwNP remains unknown.

Under hypoxic conditions, HIF‐1α mediates the transition from oxidative to glycolytic metabolism by activating pyruvate dehydrogenase kinase (PDK1) which inactivates pyruvate dehydrogenase (PDH), the mitochondrial enzyme responsible for converting pyruvate to acetyl‐CoA for entry into the tricarboxylic acid (TCA) cycle. HIF‐1α also stimulates lactate dehydrogenase A (LDHA), which converts pyruvate to lactate, and BNIP3 and BNIP3L, which induce mitochondrial autophagy.^7^ Upregulated PDK1 and subsequent lactate accumulation have been shown to facilitate EMT in alveolar epithelial cells and promote the development of pulmonary fibrosis in idiopathic pulmonary fibrosis (IPF).^8^ However, it remains unclear whether the PDK1‐lactate axis is involved in the hypoxia/HIF‐1α‐induced EMT process in CRSwNP.

In this study, we combined transcriptomic and proteomic analyses of hypoxia‐treated human nasal epithelial cells (HNECs) and identified PDK1 and LDHA as two of the most significantly upregulated glycolysis‐related enzymes in patients with CRSwNP. Furthermore, we demonstrated a positive correlation between the upregulation of PDK1 and LDHA and the occurrence of EMT in nasal tissues. We also confirmed that hypoxia may upregulate PDK1 expression, subsequently leading to EMT through lactate production in HNECs. These data provide valuable insights into the mechanisms of EMT development and potential therapeutic targets for CRSwNP treatment.

## 2 MATERIALS AND METHODS

### 2.1 Subjects

Nineteen patients with CRSwNP, diagnosed according to the European Position Paper on Rhinosinusitis and Nasal Polyps 2020 guidelines,^1^ and 18 healthy controls undergoing septoplasty for anatomical variations and without other sinonasal diseases, were enrolled in the study at Beijing Tongren Hospital. Patients with immune system disorders, fungal sinusitis, cystic fibrosis, primary ciliary dyskinesia, or severe uncontrolled systemic diseases were excluded from the study. None of the patients had received corticosteroids or antibiotics in the month prior to inclusion. ECRSwNP and NECRSwNP were defined based on the proportion of eosinophils infiltrating the nasal tissues in line with previous investigations.^9^ The clinical characteristics of all participants are presented in Supporting Information S1: Table S1. This study was approved by the Ethics Committee of Beijing Tongren Hospital, and informed consent was obtained from each patient before sample collection.

### 2.2 Cell culture

Primary HNECs were isolated from nasal polyp tissue of CRSwNP patients during endoscopic sinus surgery. Following enzymatic digestion with 0.1% protease (type XIV) (Sigma‐Aldrich, St. Louis, MO, USA), purified HNECs were seeded onto 6.5 mm‐diameter transwell inserts with a pore size of 0.4 μm (Costar, Corning, NY, USA) coated with human placental collagen, at a density of 150,000 cells per well in bronchial epithelial growth medium (BEGM). Upon reaching confluence, an air‐liquid interface (ALI) cell culture was established by removing the apical medium, and the cells were fed from the basolateral side with BEGM: DMEM (1:1) supplemented with 50 nM all‐trans retinoic acid. The culture medium was changed every other day.

The human nasal epithelial cell line (HNEpC) was cultured in DMEM supplemented with 10% fetal bovine serum at 37°C in a 5% CO_2_ atmosphere. Cells were utilized for experiments within five passages.

All cell experiments were conducted with a minimum of two replicates per condition and repeated at least three times using independent batches of cells.

### 2.3 Hypoxia treatment

Hypoxia treatment was initiated 14 days after establishment of ALI culture in primary HNECs. Hypoxic condition was produced by culturing cells in 1% O_2_, 5% CO_2_ in an anaerobic incubator at 37°C for 72 h. HNECs incubated under normoxic conditions (21% O_2_, 5% CO_2,_ 37°C) were used as controls.

### 2.4 RNA sequencing analysis

RNA was extracted from HNECs following hypoxia treatment for 72 h. RNA sequencing was carried out by Novogene (Beijing, China). RNA integrity was assessed by the RNA Nano 6000 Assay Kit of the Bioanalyzer 2100 system. Library construction, clustering and sequencing were performed according to the manufacturer's instructions. Raw data of fastq format were processed through fastp software. Clean data were obtained by removing reads containing adapters and low‐quality reads. Reference genome and gene model annotation files were downloaded from genome website directly. Index of the reference genome was built using Hisat2 v2.0.5 and paired‐end clean reads were aligned to the reference genome using Hisat2 v2.0.5. FeatureCounts v1.5.0‐p3 was used to count the reads numbers mapped to each gene. And then FPKM of each gene was calculated based on the length of the gene and reads count mapped to this gene. Differentially expressed genes (DEGs) were screened using the DESeq2 R package (1.20.0) with threshold values |log2foldchange| ≥ 1 and *p*‐value < 0.05.

### 2.5 Proteomic analysis

Total protein was extracted from the same HNECs as those used for RNA sequencing. Proteomic analysis was performed by Jingjie PTM Biolab (Hangzhou, China). Briefly, the extracted protein samples were digested in trypsin solution, and then the peptides were desalted by Strata X SPE column, followed by LC‐mass spectrometry (MS)/MS analysis. The DIA data were processed using DIA‐NN search engine (v.1.8). Tandem mass spectra were searched against Homo_sapiens_9606_SP_20230103.fasta (20389 entries) concatenated with reverse decoy database. Trypsin/P was specified as cleavage enzyme allowing up to 1 missing cleavages. Excision on N‐term Met and carbamidomethyl on Cys were specified as fixed modifications. FDR was adjusted to < 1%. Differentially expressed proteins (DEPs) were selected by |log2foldchange| ≥ 0.5 and *p*‐value < 0.05.

### 2.6 Functional enrichment analysis

Gene Ontology (GO) and Kyoto Encyclopedia of Genes and Genomes (KEGG) pathway enrichment analyses were performed on DAVID (https://david.ncifcrf.gov/), with a *p*‐value < 0.05 was considered significantly enriched.

### 2.7 Protein‐protein interaction (PPI) network

Protein–protein interaction (PPI) network was constructed by using STRING database (http://string‐db.org) and visualized by Cytoscape_3.7.2 software. The hub genes were identified by using the CytoHubba plugin.

### 2.8 Cell transfection

The PDK1‐overexpression (PDK1‐oe) plasmid, which was generated by inserting PDK1 cDNA into a pEX‐3 vector, and its empty control pEX‐3 plasmid, was provided by Gene Pharma (Suzhou, China). HNEpC were seeded in 24‐well plates at a density of 1.5 × 10^5^ cells/well, and incubated at 37°C overnight. Transfection of PDK1‐oe was performed using Lipofectamine 3000 (Invitrogen, USA) according to the manufacturer's instructions. Cells were harvested after 48 h for subsequent analysis.

### 2.9 Lactate dehydrogenase activity and lactate assay

Lactate dehydrogenase (LDH) activity and lactate level in cell supernatants were determined using the LDH activity assay kit (Yeasen, Shanghai, China) and lactate assay kit (Nanjing Jiancheng Bioengineering Institute, Nanjing, China), respectively. The corresponding operations were performed according to the manufacturer's instructions.

### 2.10 Real‐time PCR

Total RNA was extracted from cells using the RNApure Kit (Cwbio, Beijing, China) and reverse transcribed to cDNA using PrimeScriptTM RT Master Mix (Takara, Japan). Quantitative PCR was carried out using the SYBR Green assay kit (Takara, Japan). β‐actin was used as the endogenous reference for mRNAs, and relative expression was calculated by the comparative cycle threshold method. The sequences of primers are listed in Supporting Information S1: Table S2.

### 2.11 Western blotting

Total protein was extracted from cells using RIPA lysis buffer containing 1% protease inhibitor, and the protein concentration was determined with a BCA assay kit (Biyuntian, Shanghai, China). Then proteins were separated on SDS‐PAGE gels and transferred to PVDF membranes. The membranes were blocked with 5% skim milk for 1 h at room temperature, and then incubated overnight at 4°C with primary antibody including anti‐HIF‐1α (1:500, Abcam), anti‐ZO‐1 (1:1000, Abcam), anti‐E‐cadherin (1:1000, Cell Signaling), anti‐vimentin (1:5000, Proteintech), anti‐α‐SMA (1:500, Abcam), and anti‐β‐tubulin (1:10000, ABclonal), separately. Then, the membranes were incubated with the corresponding secondary antibody for 1 h at room temperature. Protein bands were detected using ChemiDocTM MP Imaging System (Bio–Rad, UK), and densitometric analysis was performed using Image Lab software 6.1.0 (Bio‐Rad, UK).

### 2.12 Immunohistochemistry (IHC)

Paraffin‐embedded nasal tissue sections were deparaffinized, hydrated, antigen retrieved, and blocked before being incubated overnight at 4°C with primary antibodies: anti‐HIF‐1α (1:300, Abcam), anti‐PDK1 (1:500, Abcam), and anti‐LDHA (1:1000, Cell Signaling). The following day, sections were incubated with the corresponding secondary antibody for 1 h at room temperature, followed by staining with diaminobenzidine (DAB) solution and hematoxylin. Images were captured using an Olympus BX1 microscope at 40X magnification, and positive cells were counted using Fiji software (https://imagej.net/software/fiji/). The number of positive cells was determined in three high power fields (40×) by two independent observers who were blinded to the group assignment.

### 2.13 Immunofluorescence (IF)

For nasal tissues, after deparaffinization, hydration, antigen retrieval, and blocking, the tissue sections were incubated overnight at 4°C with anti‐ZO‐1 (1:1000, Abcam), anti‐E‐cadherin (1:1600, Cell Signaling), anti‐α‐SMA (1:200, Proteintech), or anti‐vimentin (1:5000, Proteintech). The following day, sections were incubated with a fluorescent‐labeled secondary antibody and subsequently stained with DAPI. Imaging was performed using an Olympus IX81 confocal microscope at 40X magnification, and fluorescence intensity was assessed using Fiji software.

For cell cultures, cells were fixed, permeabilized, and blocked before incubating overnight at 4°C with anti‐PDK1 (1:500, Abcam). The remaining steps followed those used for tissue sections.

The immunofluorescence staining intensity was determined in three high power fields (40×) by two independent and blinded observers.

### 2.14 Statistical analysis

Statistical analysis was conducted using GraphPad Prism 10.2.0 software. The paired *t* test, Ratio paired *t* test or Wilcoxon test was employed to compare data between two groups, while ordinary one‐way ANOVA, Krystal–Wallis test or RM one‐way ANOVA was used to assess differences among more than two groups. Spearman correlation analysis was performed to evaluate the association between the two variables. Fisher’s exact test was utilized for qualitative data. A *p*‐value of less than 0.05 was considered statistically significant.

## 3 RESULTS

### 3.1 Transcriptome profiling of hypoxia‐treated HNECs

**FIGURE 1:**
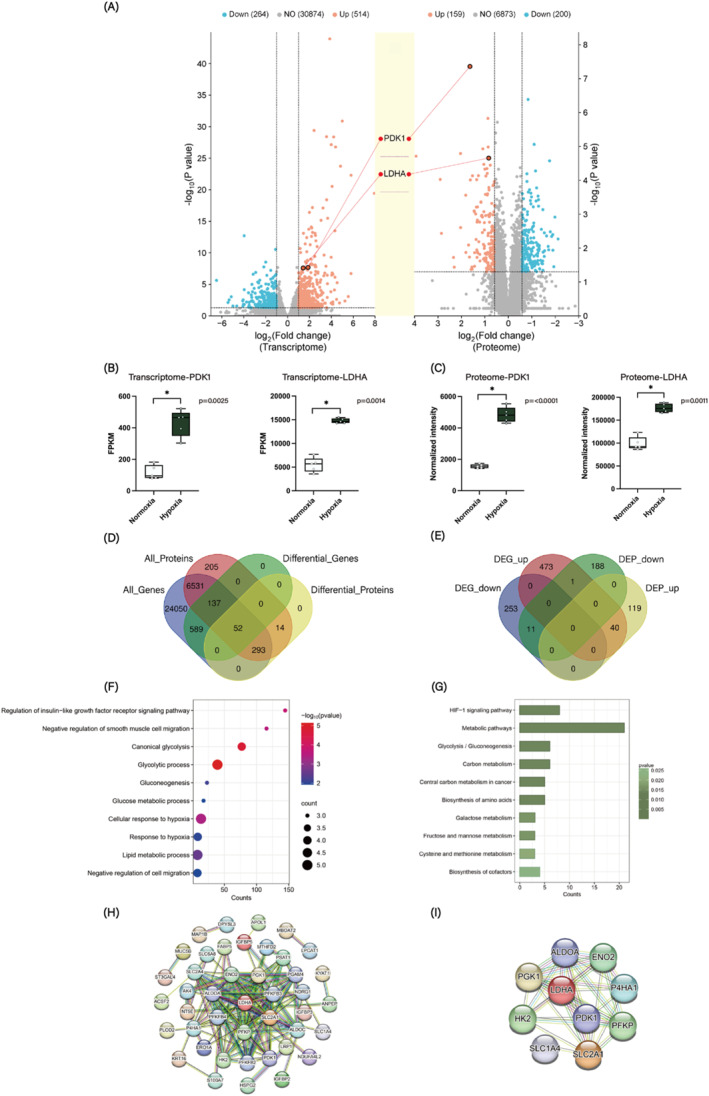
Combined analysis of transcriptome and proteome in hypoxia and normoxia groups. (A) Volcano plots illustrating DEGs/DEPs between the hypoxia and normoxia groups. (B) Expression levels of PDK1 and LDHA in transcriptome sequencing samples of hypoxia and normoxia groups. (C) Expression levels of PDK1 and LDHA in proteome sequencing samples of hypoxia and normoxia groups. (D) Venn diagrams illustrating the overlap of all detected genes/proteins and DEGs/DEPs. (E) Venn diagrams showing the overlap of upregulated and downregulated DEGs/DEPs. (F) GO enrichment analysis of overlapping DEGs/DEPs, highlighting the top 10 BP terms. (G) KEGG enrichment analysis of overlapping DEGs/DEPs, presenting the top 10 pathways. (H) PPI network of overlapping DEGs/DEPs. (I) Identification of the top 10 hub genes from the PPI network.

RNA sequencing was conducted on hypoxia‐treated HNECs. Compared to the normoxia control group, a total of 778 mRNAs were differentially expressed in the hypoxia‐treated cells, comprising 514 upregulated differentially expressed genes (DEGs) and 264 downregulated DEGs (Figure 1A). Among the DEGs, PDK1 presented a fold change of 3.67 and LDHA of 2.69, both showing a pronounced upward tendency in the sequenced samples (Figure 1A,B). GO and KEGG enrichment analyses were performed to explore the biological functions of these DEGs. A total of 112 GO biological process (BP) terms were enriched in the hypoxia group compared to the normoxia group, including “response to hypoxia,” “angiogenesis,” “regulation of glucose metabolic process,” “lipid metabolic process,” and “glycolytic process” (Supporting Information S1: Figure S1A). The KEGG pathways enriched by these DEGs were primarily associated with metabolic pathways, the HIF‐1 signaling pathway, biosynthesis of amino acids, glycolysis/gluconeogenesis, and arachidonic acid metabolism (Supporting Information S1: Figure S1B).

### 3.2 Proteome profiling of hypoxia‐treated HNECs

A total of 359 differentially expressed proteins (DEPs) were identified in the hypoxic group compared with the normoxic group, including 159 upregulated and 200 downregulated DEPs. Of these DEPs, PDK1 and LDHA manifested fold changes of 3.11 and 1.78, respectively (Figure 1A,C). GO enrichment analysis revealed that 50 BP terms, including “glycolytic process,” “glucose metabolic process,” “lipid metabolic process,” and “response to hypoxia,” were significantly enriched among the DEPs (Supporting Information S1: Figure S2A). Furthermore, KEGG pathway enrichment analysis indicated that the DEPs were predominantly enriched in pathways associated with metabolic processes, biosynthesis of amino acids, glycolysis/gluconeogenesis, and the HIF‐1 signaling pathway (Supporting Information S1: Figure S2B).

### 3.3 Combined analysis of transcriptome and proteome

Among the 778 DEGs and 359 DEPs identified between the hypoxia and normoxia groups, the expression of 40 members was upregulated at both the transcript and protein levels, while 11 members were downregulated at both levels. Notably, only one member exhibited the opposite trend (Figure 1D,E). GO enrichment analysis indicated that these common DEGs and DEPs were significantly enriched in processes related to “glycolytic process,” “cellular response to hypoxia,” “canonical glycolysis,” and “response to hypoxia” (Figure 1F). Additionally, KEGG pathway analysis revealed that the common DEGs and DEPs were enriched in “metabolic pathways,” the “HIF‐1 signaling pathway,” and “glycolysis/gluconeogenesis,” among others (Figure 1G).

To further investigate the interactions among these overlapping members, a PPI network was constructed, as shown in Figure 1H,I. A total of 43 nodes and 78 edges were identified by using STRING database (Figure 1H). The top 10 hub genes identified within the PPI network include LDHA, PGK1, SLC2A1, HK2, PFKP, ENO2, ALDOC, PDK1, SLC2A4, and P4HA1, of which 9 are involved in glucose metabolism and glycolysis (Figure 1I).

### 3.4 High expression of PDK1 and LDHA is correlated with EMT in CRSwNP

**FIGURE 2:**
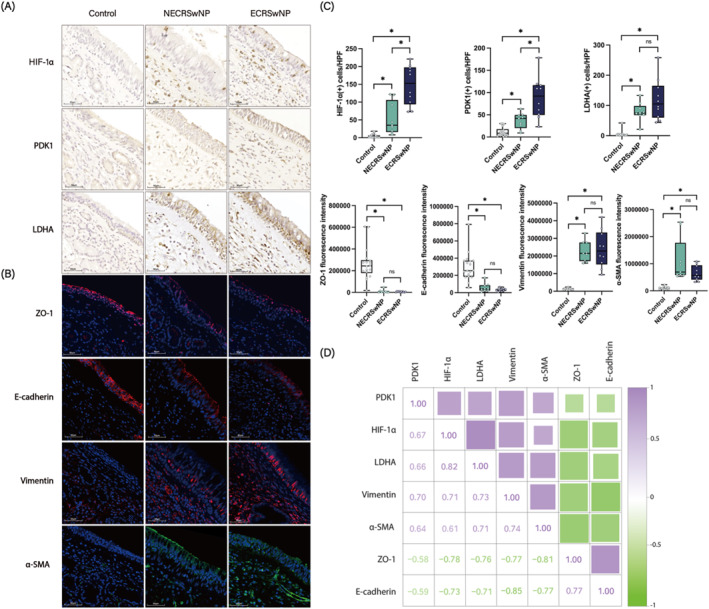
Correlation between PDK1, LDHA, and EMT in nasal tissues of CRSwNP. (A) Representative images of IHC staining for HIF‐1α, PDK1, and LDHA in control (*n* = 18), NECRSwNP (*n* = 9) and ECRSwNP groups (*n* = 10) at 40X magnification. (B) Representative images of IF staining for ZO‐1, E‐cadherin, vimentin, and α‐SMA in control (*n* = 18) NECRSwNP (*n* = 9) and ECRSwNP groups (*n* = 10) at 40X magnification. (C) Comparison of HIF‐1α (+), PDK1 (+), and LDHA (+) cell counts, as well as ZO‐1, E‐cadherin, vimentin, and α‐SMA fluorescence intensity between control, NECRSwNP and ECRSwNP groups. (D) Correlation analysis of HIF‐1α (+) cell counts, PDK1 (+) cell counts, LDHA (+) cell counts, ZO‐1 fluorescence intensity, E‐cadherin fluorescence intensity, vimentin fluorescence intensity and α‐SMA fluorescence intensity. *p < 0.05 between the two groups.

The data presented identify PDK1 and LDHA as the most significantly upregulated glycolysis‐related enzymes in hypoxia‐treated HNECs. To explore the potential relationship between hypoxia‐induced PDK1/LDHA expression and the occurrence of EMT in NP tissues, we conducted IHC and IF staining to assess the expression levels of HIF‐1α, PDK1, LDHA, and EMT markers in nasal tissues from control subjects and CRSwNP patients. Our results indicated that the number of HIF‐1α(+), PDK1(+), and LDHA(+) cells in the nasal tissues of CRSwNP patients was significantly higher than in control subjects, moreover, the numbers of HIF‐1α(+) and PDK1(+) cells in the ECRSwNP group were more higher than those in the NECRSwNP group (Figure 2A,C). Additionally, in comparison with the control tissues, the fluorescence intensities of the epithelial markers ZO‐1 and E‐cadherin were reduced, whereas the fluorescence intensities of the mesenchymal markers vimentin and α‐SMA were elevated in NP tissues (Figure 2B,C). IF costaining of PDK1 and vimentin showed a partial colocalization pattern of these two proteins, and the number of PDK1 and vimentin double‐positive cells was higher in both NECRSwNP and ECRSwNP tissues compared to control tissues (Supporting Information S1: Figure S3). These findings suggest that both hypoxia/glycolysis and EMT are critical pathological processes in NP development.

Spearman correlation analysis revealed significant correlations among the expressions of these molecules. Specifically, there was a notable positive correlation between HIF‐1α(+) cell numbers and both PDK1(+) and LDHA(+) cell numbers, as well as vimentin and α‐SMA fluorescence intensity. Similar positive correlations were observed between PDK1(+) and LDHA(+) cell numbers, vimentin and α‐SMA fluorescence intensity; and between LDHA(+) cell numbers and vimentin and α‐SMA fluorescence intensity. Conversely, HIF‐1α(+), PDK1(+), and LDHA(+) cell numbers exhibited significant negative correlations with ZO‐1 and E‐cadherin fluorescence intensity (Figure 2D). Collectively, these results suggest that the hypoxia‐induced upregulation of the glycolysis‐related enzymes PDK1 and LDHA may be linked to the occurrence of EMT in NPs.

### 3.5 Hypoxia‐induced EMT of HNECs is accompanied by PDK1 upregulation and lactate production

**FIGURE 3:**
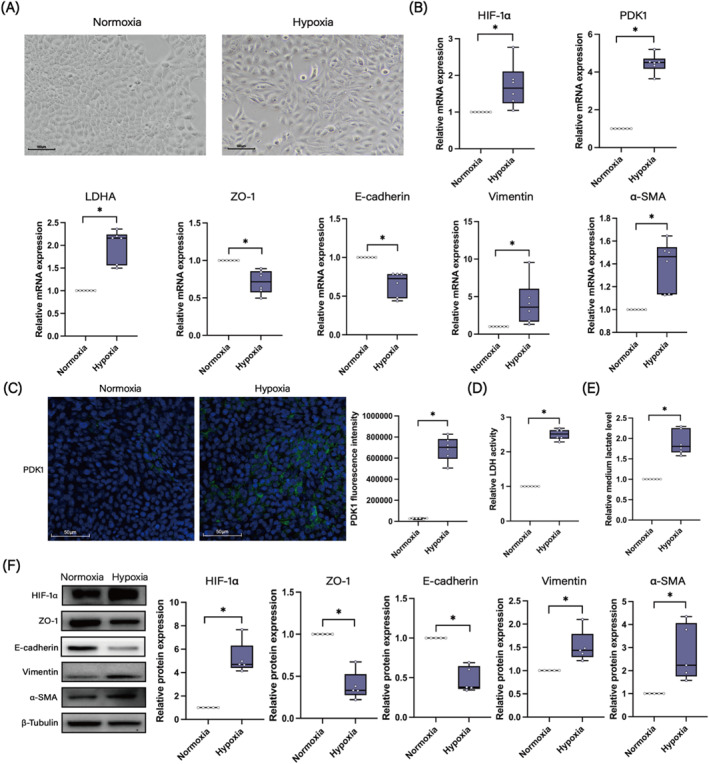
Hypoxia‐induced EMT in HNECs is accompanied by upregulation of PDK1 and LDHA, and lactate production. (A) Representative morphological changes of HNECs after 72‐h hypoxia treatment at 10X magnification (*n* = 3). (B) mRNA expression levels of HIF‐1α, PDK1, LDHA, ZO‐1, E‐cadherin, vimentin, and α‐SMA in hypoxia and normoxia groups (*n* = 6). (C) Representative images of IF staining and fluorescence intensity analysis of PDK1 in hypoxia and normoxia groups (*n* = 6) at 40X magnification. (D) LDH activity measurements in hypoxia and normoxia groups (*n* = 6). (E) Medium lactate levels in hypoxia and normoxia groups (*n* = 6). (F) Western blot analysis of HIF‐1α, ZO‐1, E‐cadherin, vimentin, and α‐SMA in hypoxia and normoxia groups (*n* = 5). *p < 0.05 between the two groups.

To explore the involvement of the PDK1‐lactate axis in hypoxia‐induced EMT of HNECs, we incubated HNECs under hypoxic conditions (1% O_2_) for 72 h and assessed the expression of PDK1, LDHA, lactate production and EMT occurrence. After hypoxic incubation, HNECs underwent remarkable morphological transition from cobblestone‐like epithelial phenotype to elongated mesenchymal‐like appearance (Figure 3A). Hypoxia treatment induced a significant increase in both mRNA and protein levels of HIF‐1α (Figure 3B,F), confirming the establishment of a hypoxic environment. Hypoxia treatment also resulted in a marked upregulation of PDK1 and LDHA mRNA expression, along with an increase in PDK1 protein levels, LDH activity, and lactate level in the media (Figure 3B–E). Concurrently, HNECs underwent a decrease in epithelial markers (ZO‐1 and E‐cadherin) and an increase in mesenchymal markers (vimentin and α‐SMA) (Figure 3B,F). These findings indicate that both the PDK1‐lactate axis and EMT are enhanced in HNECs under hypoxic conditions.

### 3.6 PDK1 leads to lactate production and promotes EMT of HNEpC

**FIGURE 4:**
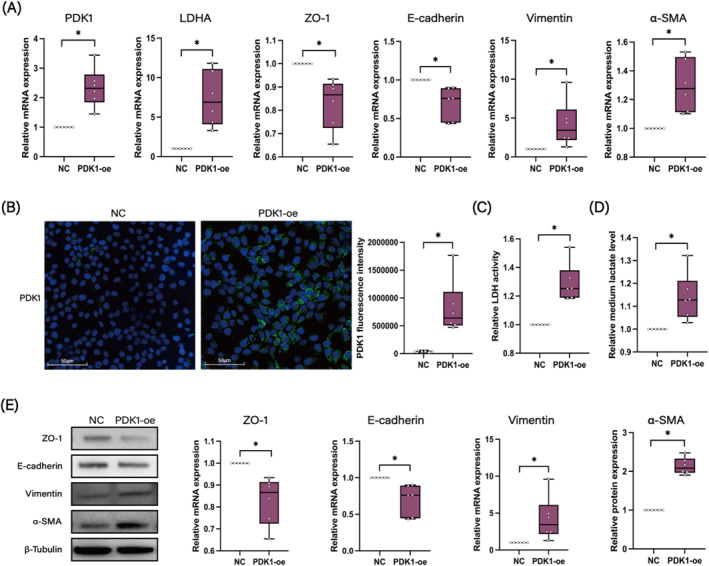
PDK1 induces lactate production and EMT in HNEpC. (A) mRNA expression levels of PDK1, LDHA, ZO‐1, E‐cadherin, vimentin, and α‐SMA in negative control (NC) and PDK1 overexpression (PDK1‐oe) groups (*n* = 6). (B) Representative images of IF staining and fluorescence intensity analysis of PDK1 in NC and PDK1‐oe groups (*n* = 6) at 40X magnification. (C) LDH activity measurements in NC and PDK1‐oe groups (*n* = 6). (D) Medium lactate levels in NC and PDK1‐oe groups (*n* = 6). (E) Western blot analysis of ZO‐1, E‐cadherin, vimentin, and α‐SMA in NC and PDK1‐oe groups (*n* = 6). *p < 0.05 between the two groups.

To verify the role of PDK1 in inducing EMT, HNEpC was transfected with PDK1 cDNA to achieve overexpression. The efficacy of this overexpression was confirmed at both the mRNA (Figure 4A) and protein levels (Figure 4B). Following PDK1 overexpression, there was a notable increase in the expression of LDHA, LDH activity, and lactate production in HNEpC (Figure 4A,C, and D). Additionally, high levels of PDK1 led to decreased expression of ZO‐1 and E‐cadherin, along with increased expression of vimentin and α‐SMA, at both the gene (Figure 4A) and protein levels (Figure 4E), indicating the occurrence of EMT. These results suggest that PDK1 overexpression may promote EMT in nasal epithelial cells, potentially through the induction of lactate production.

### 3.7 Lactate induced the EMT of HNECs

**FIGURE 5:**
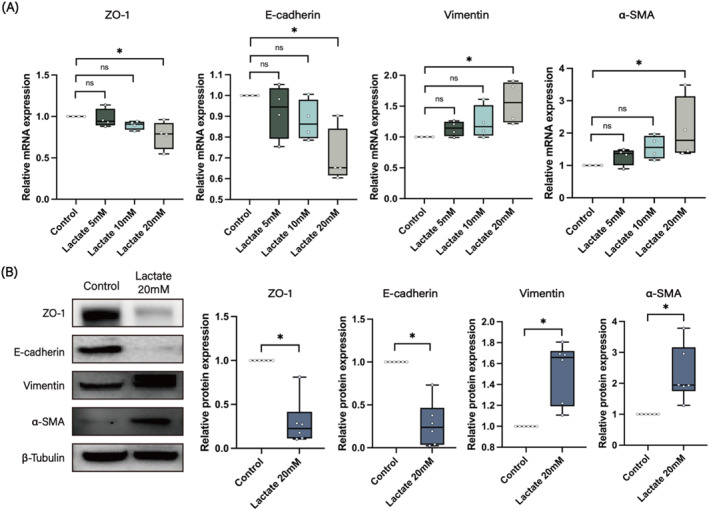
Lactate stimulation induces EMT in HNECs. (A) mRNA expression levels of ZO‐1, E‐cadherin, vimentin, and α‐SMA in HNECs after stimulation with various concentrations of lactate (*n* = 4). (B) Western blot analysis of ZO‐1, E‐cadherin, vimentin, and α‐SMA in control and 20 mM lactate‐stimulated HNECs (*n* = 6). *p < 0.05 between the two groups.

Next, we examined the role of lactate in inducing EMT in HNECs. Initially, we treated the HNECs with varying concentrations of lactate (Sigma‐Aldrich, St. Louis, MO, USA) for 24 h and subsequently harvested the cells for real‐time PCR analysis. As shown in Figure 5A, lactate treatment downregulated the mRNA expression of ZO‐1 and E‐cadherin while upregulating the mRNA expression of vimentin and α‐SMA in a dose‐dependent manner, with statistically significant differences observed at a concentration of 20 mM. Therefore, we treated the cells with 20 mM lactate for 48 h to assess the protein expression levels of EMT‐related markers. Consistent with the gene expression results, the findings revealed that treatment with 20 mM lactate suppressed the protein expression of epithelial markers such as ZO‐1 and E‐cadherin while simultaneously increasing the expression of mesenchymal markers, including vimentin and α‐SMA (Figure 5B). This confirms the effects of lactate in promoting EMT in HNECs.

### 3.8 PDK1 inhibitor attenuated the hypoxia‐induced EMT in HNECs

**FIGURE 6:**
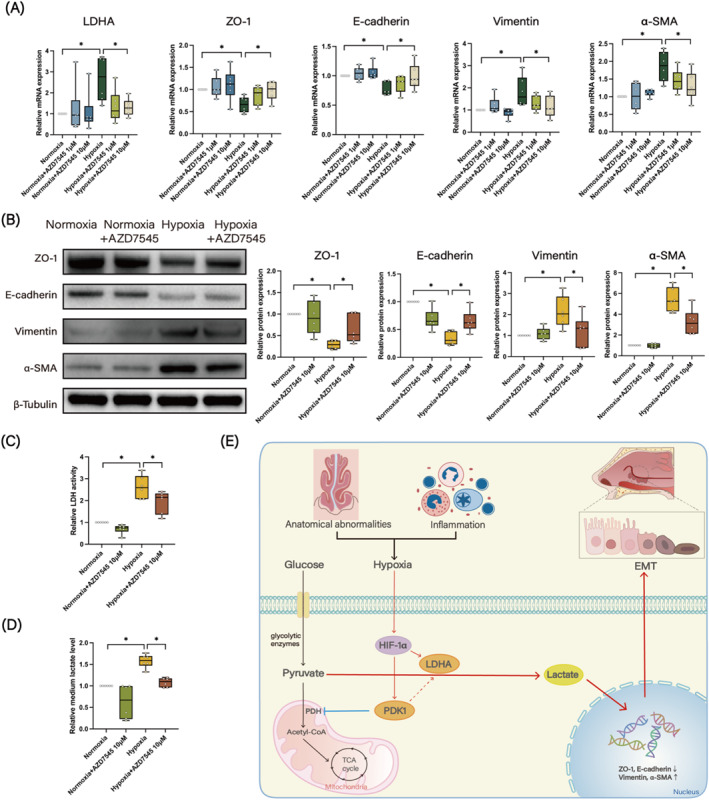
PDK1 inhibitor attenuates hypoxia‐induced EMT in HNECs. (A) Effect of different concentrations of AZD7545 on the mRNA expression levels of LDHA, ZO‐1, E‐cadherin, vimentin, and α‐SMA in HNECs subjected to hypoxia treatment (*n* = 6). (B) Effect of AZD7545 on the protein levels of ZO‐1, E‐cadherin, vimentin and α‐SMA in HNECs subjected to hypoxia treatment (*n* = 6). (C) Effect of AZD7545 on LDH activity in HNECs subjected to hypoxia treatment (*n* = 6). (D) Effect of AZD7545 on medium lactate levels in HNECs subjected to hypoxia treatment (*n* = 6). (E) Schematic diagram illustrating of the mechanism by which PDK1 regulates the occurrence of EMT in HNECs under hypoxic conditions. *p < 0.05 between the two groups.

Finally, to further investigate the role of PDK1 in EMT, we examined the effect of AZD7545, a PDK1 inhibitor, on hypoxia‐induced EMT in HNECs. HNECs were subjected to diverse concentrations of AZD7545 (1 or 10 μM, MCE, New Jersey, USA) for 72 h under either normoxic or hypoxic conditions. Subsequently, the cells were collected for further analytical procedures. The results demonstrated that treatment with 10 μM AZD7545 notably suppressed the hypoxia‐induced alterations in EMT markers, both at the mRNA and protein levels (Figure 6A,B). Additionally, the application of 10 μM AZD7545 led to a decrease in LDHA expression, LDH activity, and the lactate level in the medium caused by hypoxia (Figure 6A,C and D). Collectively, these results corroborate the role of PDK1 in regulating hypoxia‐induced EMT in HNECs.

## 4 DISCUSSION

CRSwNP is a heterogeneous condition triggered by a combination of genetic, anatomical, and environmental factors.^10^ Considerable evidence suggests that hypoxic conditions significantly contribute to the development of CRSwNP^11^; however, the mechanisms and relevant signaling pathways involved in hypoxia's effects on CRSwNP remain largely unknown. In this study, through a combined transcriptomic and proteomic analysis, we discovered that glycolytic signaling pathways were significantly enriched in hypoxia‐treated primary HNECs, and the glycolysis‐related enzymes PDK1 and LDHA were among the most significantly upregulated molecules. Additionally, we observed a close correlation between the expression of PDK1 and LDHA and those of HIF‐1α and EMT markers in the nasal tissues of CRSwNP patients, indicating a potential role of PDK1 and LDHA in mediating hypoxia‐induced EMT in CRSwNP. Mechanistically, upregulated PDK1 can inhibit the activity of PDH, thus reducing the amount of pyruvate entering the TCA cycle in mitochondria, as a result, more pyruvate accumulates within cell and is subsequently converted into lactate under the action of LDHA (as depicted in Figure 6E). Given that the potential role of lactate in promoting EMT has been demonstrated in diseases such as myocardial infarction and diabetic nephropathy,^12^, ^13^, ^14^ we hypothesized the involvement of the PDK1 in mediating the EMT of HNECs via lactate under hypoxic conditions and tested this hypothesis using in vitro cultured HNECs. Our results showed that lactate stimulation indeed promotes the development of EMT in HNECs. Furthermore, overexpression of PDK1 in HNEpC enhanced lactate production and EMT development, while inhibiting PDK1 significantly suppressed hypoxia‐induced lactate production and EMT development. Therefore, our findings propose that the PDK1‐lactate axis mediates hypoxia‐induced EMT in CRSwNP, providing a new potential therapeutic target for this disease.

The hypoxia‐induced EMT process has been shown to contribute to nasal polyposis in CRSwNP, mediated by HIF‐1α and phosphorylated Smad3 (pSmad3), leading to a loss of E‐cadherin and induction of α‐SMA.^5^ Additionally, sirtuin 1 (SIRT1), a histone deacetylase that suppresses the transcriptional activity of HIF‐1, has been reported to inhibit HIF‐1‐induced EMT, thereby attenuating nasal polyposis.^15^ However, beyond these studies, the mechanisms underlying hypoxia‐induced EMT in CRSwNP remain poorly understood. Serving as a key glycolysis‐related enzyme downstream of HIF‐1α during hypoxic conditions, the role of PDK1 in regulating EMT has been documented in various cancers. For instance, Xie et al. identified PDK1 as a novel direct target of miR‐148a, and its increased expression reversed the EMT‐suppressive effects of miR‐148a, thereby promoting EMT.^16^ Similarly, Mao et al. suggested that PDK1, as a key downstream target of the IRE1α‐XBP1 pathway, effectively promotes EMT in non‐small cell lung cancer.^17^ Zhang et al. demonstrated that the enhanced EMT process associated with cisplatin resistance in ovarian cancer was strongly linked to increased phosphorylation of epidermal growth factor receptor (EGFR) due to PDK1 overexpression.^18^ Furthermore, PDK1 has been implicated in the EMT process in pulmonary fibrosis through its role in facilitating lactate accumulation via glycolysis.^8^ Thus, elucidating the role of the PDK1 in the regulation of EMT may offer potential therapeutic avenues for CRSwNP. In this study, we demonstrate that PDK1 is upregulated in the nasal mucosa of CRSwNP patients and is correlated with HIF‐1α and EMT markers. In in vitro experiments, we show that PDK1 overexpression leads to the occurrence of EMT in human nasal epithelial cells, whereas PDK1 inhibition suppresses hypoxia‐induced EMT in these cells. These findings indicate that hypoxia‐induced PDK1 expression is closely associated with EMT development in CRSwNP.

Our results also identify lactate as a direct mediator by which PDK1 induces EMT in HNECs. Recent studies have demonstrated that lactate is no longer viewed merely as a metabolic waste product; rather, it functions as an important regulator of various pathological and physiological processes. For instance, lactate has been reported to play a critical role in promoting chemotherapy resistance in colorectal cancer.^19^ Additionally, it has been shown to exacerbate myocardial infarction and cardiac dysfunction by facilitating endothelial‐to‐mesenchymal transition (EndoMT).^12^ PDK1‐facilitated lactate accumulation has also been implicated in promoting pulmonary fibrosis through the regulation of trans‐differentiation in lung fibroblasts and EMT in alveolar epithelial cells.^8^ Moreover, lactate may serve as a potential biomarker for the risk of kidney disease progression, as its accumulation in the kidneys can promote EMT and contribute to kidney injury in diabetic nephropathy.^13^, ^14^ In this study, we provide direct evidence that PDK1‐enhanced production of lactate, the end product of glycolysis under hypoxic conditions, might function as a key mediator in regulating hypoxia‐induced EMT in HNECs of CRSwNP.

In addition to PDK1, the present study highlights the role of LDHA, another glycolysis‐related enzyme, in regulating hypoxia‐induced EMT in NPs. IHC staining of human polyp tissue sections revealed that LDHA expression was elevated compared to control tissues and significantly correlated with HIF‐1α and EMT markers. Correspondingly, in hypoxia‐treated HNECs, both the expression level of LDHA and the activity of LDH were significantly increased, coinciding with the occurrence of EMT. This phenomenon likely arises from LDHA's role in catalyzing the conversion of pyruvate to lactate under hypoxic conditions. Indeed, the HIF‐1α‐induced activation of LDHA and its facilitation of lactate production have been reported in various diseases, such as pancreatic cancer,^20^ supporting the findings of our study. Notably, we observed increased LDHA expression and LDH activity in PDK1‐overexpressed human nasal epithelial cells, whereas inhibition of PDK1 suppressed hypoxia‐induced LDHA expression and LDH activity. This suggests that LDHA not only acts as a downstream target of HIF‐1α but may also be regulated by PDK1 to enhance lactate production during hypoxia. A similar connection between PDK1 and LDHA has been noted in previous studies on pulmonary fibrosis^8^ and multiple myeloma^21^; however, the specific mechanisms warrant further investigation.

Collectively, the data from the current study demonstrated that PDK1 plays a crucial role in mediating the EMT in HNECs of CRSwNP via lactate under hypoxic conditions. The effect and underlying mechanism can be elucidated as follows (Figure 6E): Under hypoxic conditions, glycolysis‐related enzymes PDK1 and LDHA were significantly upregulated in HNECs. The upregulated PDK1 may inhibit PDH, thereby preventing the conversion of pyruvate into acetyl‐CoA within the mitochondria. Instead, under the catalytic action of LDHA, PDK1 promotes the conversion of pyruvate into lactate. The overproduced lactate then acts as a key driver in promoting the EMT of HNECs.

However, it must be acknowledged that several limitations exist in our experiments. Firstly, although our study uncovered a possible role of LDHA in the PDK1‐lactate axis, the precise effect and mechanism of LDHA remain to be clarified in future studies. Secondly, future studies incorporating interrelated gene interference techniques, specifically targeting HIF‐1α, PDK1, and LDHA, hold the promise of fortifying the logical chain of our findings. Thirdly, in vitro experiments cannot fully replicate in vivo conditions, necessitating further validation of these results in future animal models.

## 5 CONCLUSION

In our present study, we identify PDK1 and LDHA as key glycolysis‐related genes induced during hypoxia in CRSwNP through the combined analysis of transcriptomic and proteomic data. Based on this evidence, we propose a novel mechanism in which hypoxia induces the EMT of HNECs via the PDK1‐lactate axis. These findings suggest that inhibiting PDK1 may represent a new therapeutic approach for the treatment of CRSwNP. Further in vivo experiments are necessary to validate these results and explore their potential therapeutic implications.

## AUTHOR CONTRIBUTIONS

**Sicen Pan**: Investigation; methodology; writing—original draft. **Mengyan Zhuang**: Investigation; writing—original draft. **Xiangdong Wang**: Writing—review and editing; methodology. **Qinqin Zhang**: Methodology; investigation. **Ting He**: Investigation; data curation. **Ying Li**: Investigation; methodology. **Jian Jiao**: Conceptualization; writing—review and editing; supervision. **Luo Zhang**: Conceptualization; supervision; project administration; writing—review and editing.

## CONFLICT OF INTEREST STATEMENT

The authors declare no conflicts of interest.

## Supporting information

Supporting Information S1

## Data Availability

The data that support the findings of this study are available from the corresponding author upon reasonable request.
