# Supplementary material for: Targeting PDK1: A novel approach to combat hypoxia‐induced epithelial‐mesenchymal transition in chronic rhinosinusitis with nasal polyps
Source: Clin Transl Allergy. 2025 Apr 2;15(4):e70048. doi: 10.1002/clt2.70048 (PMC11964949; doi:10.1002/clt2.70048)

Table S1.The clinical characteristics of subjects

|  | Control group | NECRSwNP group | ECRSwNP group | P value |
| --- | --- | --- | --- | --- |
| Numbers | 18 | 9 | 10 | - |
| Age(years), mean±SD | 49.4±14.9 | 48.9±15.4 | 47.1±10.9 | 0.8266 |
| Sex(male/female) | 10M/8F | 6M/3F | 5M/5F | 0.8361 |
| AR | 0/18 | 1/9 | 3/10 | 0.0741 |
| Asthma | 0/18 | 3/9 | 1/10 | 0.0516 |
| AERD | 0/18 | 0/9 | 0/10 | >0.9999 |
| Tissue Eos(%), mean±SD | 1.9±0.02 | 2.4±0.02 | 40.1±0.10 | <0.0001 |

Abbreviation: NECRSwNP: non-eosinophilic chronic rhinosinusitis with nasal polyp; ECRSwNP: eosinophilic chronic rhinosinusitis with nasal polyp; AR, allergic rhinitis; AERD, aspirin-exacerbated respiratory disease.

Table S2. Primer sequences for real-time PCR

| Genes | Forward primer (5′-3′) | Reverse primer (5′-3′) |
| --- | --- | --- |
| HIF-1α | GCAGAATGCTCAGAGGAAGC | ACGTTCCAATTCCTGCTGCT |
| PDK1 | GATGTGAATGGGCAGTTAGT | AGGAATAGTGGGTTAGGTGAG |
| LDHA | GGTTGTGCATGTGTGTCTTCT | CGTCAGAGGTGGCAGAA |
| ZO-1 | CGGTCCTCTGAGCCTGTAAG | GGATCTACATGCGACGACAA |
| E-cadherin | TCTTCGGAGGAGAGCGGTGGTCAAA | GCCGAGCGTCCAGGCCCCTGTGCAG |
| Vimentin | CCGAAAACACCCTGCAATCTTTC | CACATCGATTTGGACATGCTGT |
| α-SMA | ATCAAGGAGAAACTGTGTTATAG | GATGAAGGATGGCTGGAACAGGGTC |
| β-actin | GATCCACATCTGCTGGAAGG | AAGTGTGACGTTGACATCCG |

Abbreviation: HIF-1α: hypoxia inducible factor-1α; PDK1: pyruvate dehydrogenase kinase 1; LDHA: lactate dehydrogenase A; ZO-1: zonula occludens-1; α-SMA: alpha smooth muscle actin.

**Figure legends:**

Figure S1. Functional enrichment analysis of differentially expressed genes (DEGs) in hypoxia and normoxia groups. (A) GO enrichment analysis of DEGs, displaying the top 30 BP terms. (B) KEGG enrichment analysis of DEGs, highlighting the top 20 pathways. (n=5)


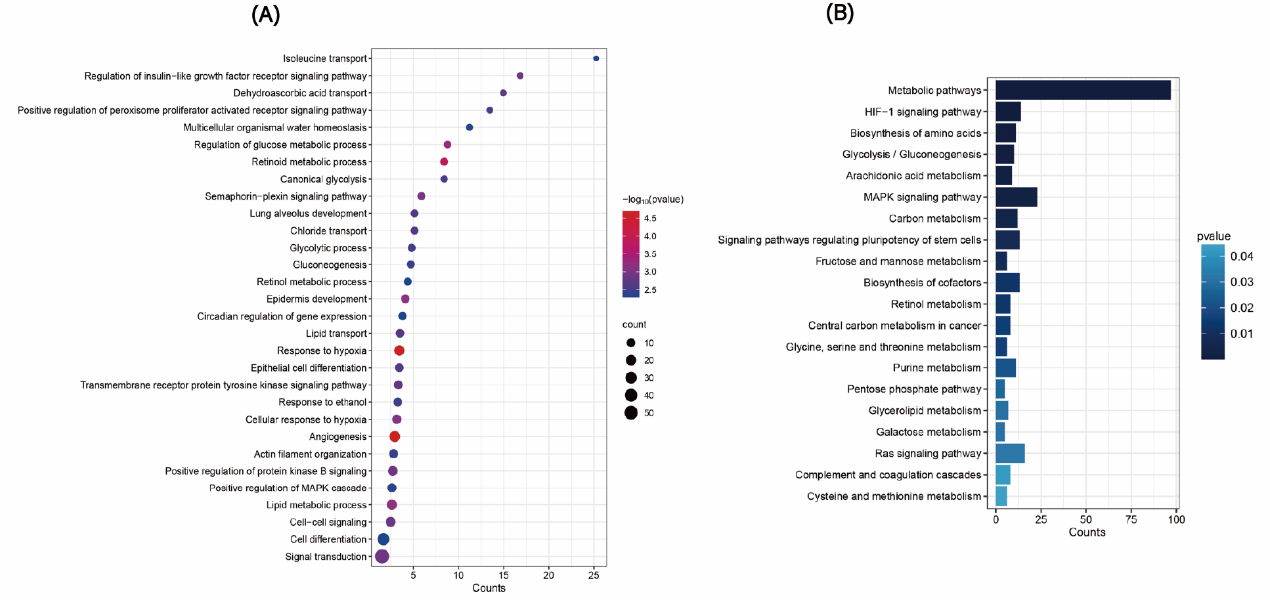


Figure S2. Functional enrichment analysis of differentially expressed proteins (DEPs) in hypoxia and normoxia groups. (A) GO enrichment analysis of DEPs, presenting the top 30 BP terms. (B) KEGG enrichment analysis of DEPs, showing the top 20 pathways. (n=5)


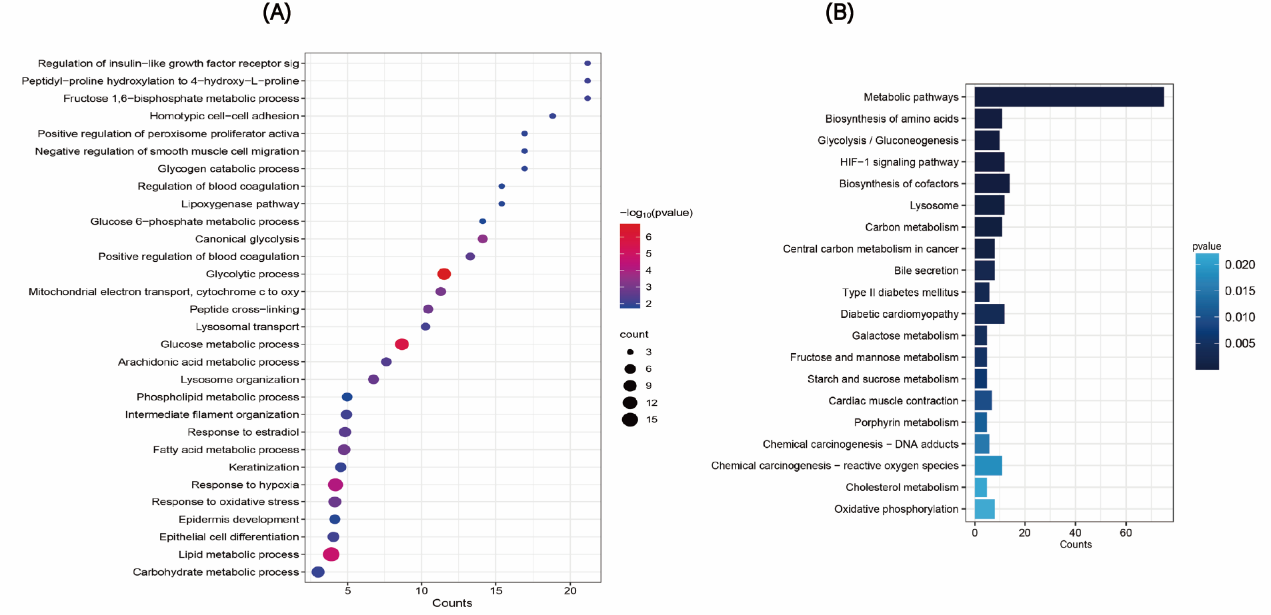


Figure S3. Colocalization of PDK1 and vimentin in nasal tissues of CRSwNP. (A) Representative images of the immunofluorescence costaining of PDK1 and vimentin in control (n=18), NECRSwNP (n=9), and ECRSwNP groups (n=10) at 40X magnification. (B) Comparison of PDK1 and vimentin double-positive cell numbers between control, NECRSwNP and ECRSwNP groups. *P < 0.05 between the two groups.


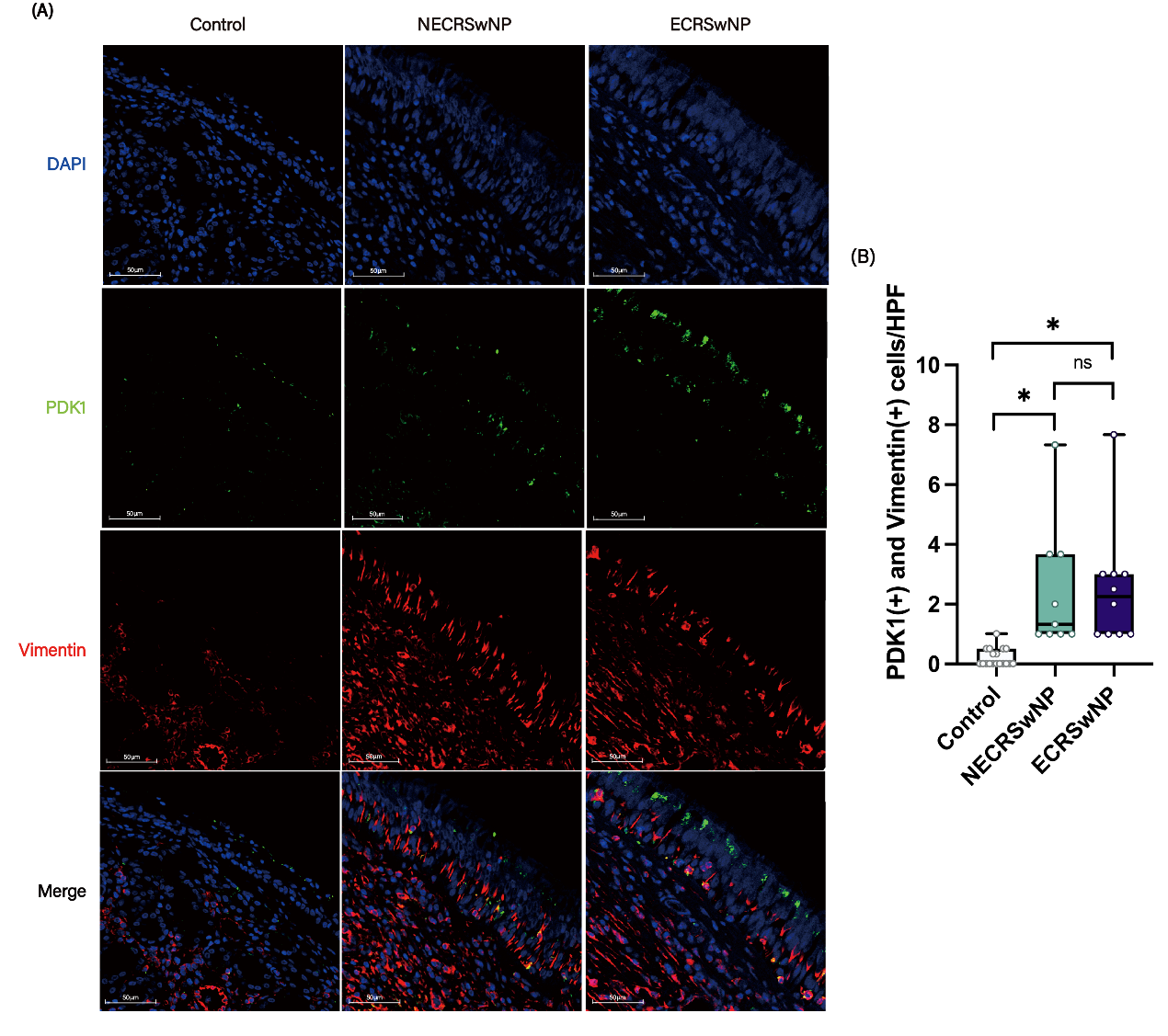

Supplement: Supplementary file 1 — Supporting Information S1 [file CLT2-15-e70048-s001.docx]
